# How Labor Values Affect Mental Health: An Analysis From the Perspective of Social Support

**DOI:** 10.3389/fpsyg.2021.783569

**Published:** 2021-12-16

**Authors:** Yuliang Gu, Xiaomei Chao

**Affiliations:** ^1^Department of Sociology, Hunan Normal University, Changsha, China; ^2^Department of Education, Hunan Normal University, Changsha, China

**Keywords:** labor values, psychological distress, life satisfaction, social support, mental health

## Abstract

To explore the positive and negative effects of labor values on mental health from the aspects of life satisfaction and psychological distress, and further verify the mediating role of social support. A total of 2,691 primary and secondary school students were surveyed by Labor Values Scale, the Multidimensional Scale of Social Support, General Health Questionnaire and Satisfaction with Life Scale, and the results of which showed that as: (1) labor values can positively predict life satisfaction, while they are negatively correlated with psychological distress; (2) social support can play a mediating role between labor values and life satisfaction; and (3) social support can also play a mediating role in the relationship between labor values and psychological distress. This study revealed that the specific path and mechanism of labor values on mental health. This provided a reference for families and schools to further implement the education of labor values on primary and secondary school students and helped to promote the social construction of an education system that aimed at cultivating individual all-round development.

## Introduction

Values are the concept system that people use to distinguish good from bad, beauty from ugliness, profit from loss, right from wrong, or to judge whether something is in line with their own wishes, etc. (Zhang, 2002; Huang and Zheng, 2015). Values are closely related to mental health (Balzarotti et al., 2016; Rean and Shagalov, 2018; Yurim et al., 2018). Labor values are the abstraction of individuals subjective evaluations on labor (Hao, 2014; Tan, 2019a), which are one of the most fundamental values derived from labor practice. Labor values are not just people’s general and fundamental views on the state and extent to which labor satisfies people’s needs, which are also the internal needs of individuals, and it can present the labor characteristics or attributes that they pursue in their activities (Guo and Liu, 2016). Therefore, labor values are of great significance in the development of human society.

In the Chinese institutional culture guided by Marxism, labor values have special meanings. According to the research of Tan (2019b), in Chinese culture, labor values refer to the individual’s positive labor attitude, that is, refusing to be lazy, hurt others, be self-interest, and own other negative values. At the same time, it encouraged and cultivated people to respect and enjoy the labor process and labor results, and to treat employees and labor results equally. The Chinese official government has also discussed what labor values should be established on many occasions. For example, President Xi Jinping believes that positive labor values should be established with the main content of honest labor, equal labor, cherishing labor results, love of labor, and distribution of benefits according to labor (Xi, 2015).

However, in recent years, the unique educational value of labor has been neglected to a certain extent, and labor education has gradually faded away, thus leading to part of the young people unwilling to work. In response to this situation, the Chinese government even issued an official document on labor education, which entitled *Opinions on comprehensively strengthening labor education in schools and colleges in the new era*, to emphasize the importance of labor education to the publics. It is suggested that labor education should be incorporated into the whole process of personnel education and integrated with moral education, intellectual education, physical education, and esthetic education, so as to promote students to establish a correct outlook on life, world, and values, and further to improve students’ physical and mental health.

Nevertheless, up to now, existing research on the interpretation of the relationship between labor values and mental health is still not comprehensive and in-depth. What has been found is the relationship between work values and mental health (Deci and Ryan, 2000; Yang et al., 2019), but the relationship and mechanism between labor values and individual physical and mental health are still unclear. Based on this, the present study intended to reveal the positive and negative effects of labor values on the mental health of primary and secondary school students from the aspects of life satisfaction and psychological distress. To discuss the relationship between labor values and mental health, we should pay attention to the role of social support. In previous studies, many scholars found that values are an important variable for predicting social support (Xiang et al., 2018; Ren et al., 2019), and social support is an important element that cannot be ignored in individual psychological development (Constantine, 2006; Feng et al., 2012). Social support mainly affects mental health through two mechanisms, namely, the main-effect model and the buffering model. The main-effect theory holds that social support has a direct enhancing effect on individual mental health, and the higher the level of social support is, the higher the level of individual mental health is (Kawachi and Berkman, 2001; Alipour, 2006). According to the buffering effect theory, social support does not directly affect individual mental health but buffers the negative impact of external adversity on individual mental health by influencing individual cognition (Cohen and Wills, 1985; Che et al., 2018). Based on this, it is necessary for us to introduce social support variables while discussing the impact of labor values on individual mental and physical development—mental health. We need to explain from the perspective of social support why labor values affect psychological distress and life satisfaction, so that labor values affect the physical and mental development of individuals.

Besides, since previous studies have emphasized the differences in values and labor participation between men and women (Duffy and Sedlacek, 2007; Hagström and Kjellberg, 2007), we hope to compare whether there are differences in labor values between boys and girls. At the same time, we also want to test the robustness of the model through comparative analysis across gender.

## Labor Values and Psychological Distress

As a subjective factor, values are of great importance to indicate individual mental health (Richins and Dawson, 1992; Liliana and Nicoleta, 2014; Balzarotti et al., 2016). And the clarity of values is positively correlated with mental health status, that is, the higher degree of clarity on values, the better mental health level of individuals (Yurim et al., 2018). Of course, there is a complex relationship between different forms of values and mental health. Positive values can significantly inhibit individuals’ negative mental health problems (Belk, 1984; Richins and Dawson, 1992). Studies on adolescents have found that adolescents with collectivist values have significantly lower anxiety levels than other adolescents (Sawrikar and Hunt, 2005; Rean and Shagalov, 2018). And studies on female college graduates indicated that those who with Taoist values, such as being open-minded and letting nature, take its course can effectively relieve their anxiety and depression (Luo and Cheng, 2015). The more the college students able to accept traditional Chinese values, the less symptoms of various psychological problems they have (Tong et al., 2010).

Labor is an important variable that requires special attention, which can affect people’s mental health (Kleiner et al., 2015; Liu et al., 2018; Kotera et al., 2020). According to Marx and Engels (2009), labor is an important way to create values, and it is also an important premise to create labor happiness. People with different values have different mental health status (Deci and Ryan, 2000; Yang et al., 2019). Chao and Wang (2020) found that positive labor value orientation is significantly negatively correlated with anxiety, depression, and psychological stress. These studies revealed that labor values have significant effects on restraining psychological distress. Therefore, we hypothesized that labor values could have a negative impact on individual psychological distress.

## Labor Values and Life Satisfaction

Life satisfaction refers to people’s overall judgments on their own life experiences, and an overall evaluation of his or her life quality according to the standard that they chose (Shin and Johnson, 1978; Yang and Ye, 2018), which is a very important positive indicator to measure individual mental health. Previous studies have found that values are important factors affecting individual life satisfaction. For example, the value orientation of materialism has a reverse prediction effect on individual life satisfaction (Hu and Dong, 2020; Ma and Ding, 2020), namely, the stronger an individual’s materialistic value is, the lower his life satisfaction would be. A comparative study of happiness in five regions of East Asia in the past two decades showed that although there are significant differences in income levels among residents in different regions, the overall difference in life satisfaction is not as large as that in income (Lim et al., 2020). The possible explanation here is that the more positive a person’s material orientation is, the more readily he or she can deal with various situations in life, and the higher his or her life satisfaction will be, which is also evident in migrant workers. For the migrant workers, their cognition and understanding of labor, especially their insistence on creating wealth and a better life through labor, are an important basis for them to maintain a high degree of life satisfaction (Gu, 2017). Therefore, it can be referred that positive labor value orientation may be closely related to higher life satisfaction. Thus, we hypothesized that labor values could have a positive impact on individual life satisfaction.

## Labor Values, Social Support, Psychological Distress, and Life Satisfaction

Although values are closely related to mental health, the underlying mechanism of how values affect mental health is still unclear. Particularly, how labor values affect mental health remains to be explored deeply. Among the factors affecting individual mental health, social support is an important variable. Social support refers to the material and spiritual support that individuals feel from family members, friends, and colleagues when they are under pressure (Yang, 2006). According to different criteria, social support can be divided into explicit social support and implicit social support, objective support and subjective support, material support and emotional support, etc. (Wills, 1991; Taylor et al., 2007). From the perspective of social support theory, social support can effectively relieve personal pressure, get individuals out of trouble, then improve their physical and mental health (Cohen and Wills, 1985; Zeng, 2011; Lu and Wu, 2019). Previous studies have found that people with positive values usually leave others a good impression and give them positive feedback, which makes them be more likely to get recognition and help from others (Feng et al., 2012; Xiang et al., 2018). What’s more, Ren et al. (2019) directly found that people with positive values are more sensitive to interpersonal relationships and pay more attention to maintain good interpersonal relationships, thus gaining more social support.

The relationship between social support and mental health is relatively complex. On the one hand, according to the buffering effect theory, social support can help individuals relieve adverse emotions and reactions caused by pressure in life, thus promoting individual mental health (Zhang et al., 2019; Dai and Fang, 2020). More specifically, not only the comfort, care and material support obtained from peer groups, but also the psychological support based on group psychotherapy can significantly improve the mental health of individuals (Cohen and Wills, 1985; Fu and Kai, 2018). And for children, the greater the interpersonal support from the environment, the more likely it is to protect children’ s mental health (Jiang and He, 2019).

On the other hand, there are also some relationships between social support and individual life satisfaction. Both objective and subjective support have a positive impact on individual life satisfaction (Song and Fan, 2013). For example, the research on college students found that the higher the material support from the outside world, the stronger the life satisfaction of college students (Ma and Wang, 2013; Miao et al., 2020). However, social support not only directly affects life satisfaction, but also indirectly affects life satisfaction through self-perception. The investigation on the life satisfaction of children who lived with their parents that were migrant workers, the results showed that social support had a significant positive predictive effect on the life satisfaction of these children and that self-cognition and self-evaluation were significantly correlated with life satisfaction (Jiang and Liu, 2016).

Since values have a close relationship with social support, social support has a significant effect on restraining psychological distress. Therefore, it is reasonable to predict that social support may mediate the effect of values on mental health. Although there is no direct evidence that labor values can influence mental health through the mediation of social support, some analyses did indicate that labor values can significantly influence social support (Lan and Liang, 2019). So we hypothesized that labor values may influence individual mental health through the mediating effect of social support.

Based on a review of existing studies, this study would explore the effects of labor values on mental health from the aspects of life satisfaction and psychological distress, and we would also investigate the mediating role of social support. The hypotheses of this study are in the following: H1: Labor values could have a negative impact on individual psychological distress; H2: Labor values could have a positive impact on individual life satisfaction; and H3: Social support could play a mediating role in the relationship between labor values and mental health in the aspects of life satisfaction and psychological distress.

## Materials and Methods

### Participants

Two primary schools, two junior middle schools, and one senior high school in a northeast city of China were selected by the method of cluster sampling, and a total of 2,749 students participated in the survey. Except for those who did not complete the questionnaire or had obvious problems with the answers, the effective sample was 2,691 with effective recovery rate of 97.89%. The participants were ranged from 9 to 18years old (*M*_age_=12.50, SD=2.00, 44.1% female), including 1,303 primary school students from grade four to grade six, 673 junior high school students, and 715 senior high school students. The specific process of the investigation is as follows: firstly, obtain the school’s consent, and the school informs the teacher or parent to obtain the informed consent. On this basis, 40min are reserved for questionnaire surveys. Researchers participate in the whole measurement process and use standardized instruction. For the sample of elementary school students, based on the consent of the survey respondent, the teacher and parents of the class must agree, while for junior high school students and high school students, the consent of the individual is required. The research was approved by the ethics committee of the researcher’s unit.

### Measurement

#### Labor Values

This study adopted the Labor Values Scale compiled by Chao and Wang (2020), which contains 15 items. It was constructed from five dimensions of labor, such as honesty, equality, cherish, love, and distribution. Sample items are like “I enjoy the process of labor” and “I respect laborers of all professions equally, whether they are cleaners or engineers,” which were scored by a 5-point Likert scale from 1 “strongly disagree” to 5 “strongly agree.” The higher labor values are indicated by higher scores. Since the scale was compiled based on the senior grade of primary school, confirmatory factor analysis was used in this study to explore the reliability of the scale in middle and high school groups. The confirmatory factor analysis indexes were as follows: *χ*^2^/df=7.118, *p*<0.001; GFI=0.93, CFI=0.92, RMSEA=0.076, SRMR=0.0583, which showed that all the indexes meet the requirements of measurement. The Cronbach’s alpha coefficient of the scale was 0.832.

#### Social Support

The Multidimensional Scale of Social Support compiled by Zimet et al. (1988) was adopted, which contains 12 questions, and divided into three supporting sources: family, friends, and significant others. It is a 7-point scale from 1 “strongly disagree” to 7 “strongly agree,” and the higher perceived social support is indicated by higher scores. The Chinese version of the Perceived Social Support Questionnaire was used, which has been demonstrated to be reliable and valid in Chinese population (Zhao et al., 2013; Xiang et al., 2020). The Cronbach’s alpha coefficient of the scale was 0.934.

#### Psychological Distress

The Chinese version of the General Health Questionnaire was employed to evaluate psychological distress, which has been demonstrated to be reliable and valid in Chinese population (Lai and Chan, 2002). It is a 4-point Likert scale from 1 “never” to 4 “always,” consisting of 12 questions and six of which are reverse scoring questions. Sample item is like “Lost much sleep over worry.” Higher scores reveal worse mental health. In this study, the Cronbach’s alpha coefficient of the scale was 0.844.

#### Life Satisfaction

The Satisfaction with Life Scale was applied to measure life satisfaction (SWLS, Diener et al. 1985), which has five items. One sample item is “If I could live my life over, I would almost change my life nothing.” All items are answered by a 7-point Likert scale from 1 “strongly disagree” to 7 “strongly agree.” The higher level of life satisfaction is reflected by higher scores. The Chinese version of SWLS has been demonstrated to be reliable and valid in Chinese people (Feng et al., 2012; Zhao et al., 2014). The Cronbach’s alpha coefficient was 0.824 in this study.

#### Analytic Strategy

Using SPSS 20.0 and AMOS 22.0 for data analysis. Firstly, perform descriptive statistics and related analysis on all variables in SPSS 20.0; secondly, build a structural model in AMOS 22.0 based on the research hypothesis to analyze the fit of the model; finally, use the Bootstrap method to further test social support. The mediating role between labor values and mental health.

## Results

### Measurement Model

Four latent variables (e.g., labor values, social support, psychological distress, and life satisfaction) and 14 observed variables were contained in the measurement model. The data indicated a good fitness of the measurement model: *χ*^2^(48, 2,691)=612.57, *p*<0.001; RMSEA=0.066; SRMR=0.038; CFI=0.963. All the factor loadings for the indicators on the latent variables were significant (*p*<0.001), indicating that all latent variables were well represented by their indicators. Table 1 showed that all the investigated variables in the model were significantly associated (*p*<0.001).

**Table 1:** Descriptive statistics and bivariate correlations for all measures.

	*M*	*SD*	1	2	3	4	5	6
1.Sex			1					
2.Age	12.50	2.00	−0.06^**^	1				
3.LV	59.03	8.65	0.06^**^	−0.17^***^	1			
4.SS	23.13	6.95	−0.01	−0.00	0.47^***^	1		
5.PD	24.03	5.98	0.11^**^	0.12^***^	−0.36^***^	−0.52^***^	1	
6.LS	23.13	6.95	−0.00	−0.18^***^	0.43^***^	0.68^***^	−0.55^***^	1

** *p<0.01*;

*** *p<0.001*.

*LV, labor value; SS, social support; LS, life satisfaction; and PD, psychological distress. Boy=1; girl=0*.

**Table 2:** Standardized indirect effects and 95% confidence intervals.

Pathways	Estimate	95% Confidence Interval	
		Lower	Upper
LV→SS→LS	0.40	0.3736	0.4365
LV→SS→PD	−0.30	−0.3411	−0.2719

*LV, labor value; SS, social support; LS, life satisfaction; and PD, psychological distress*

### Structural Model

**Figure 1:**
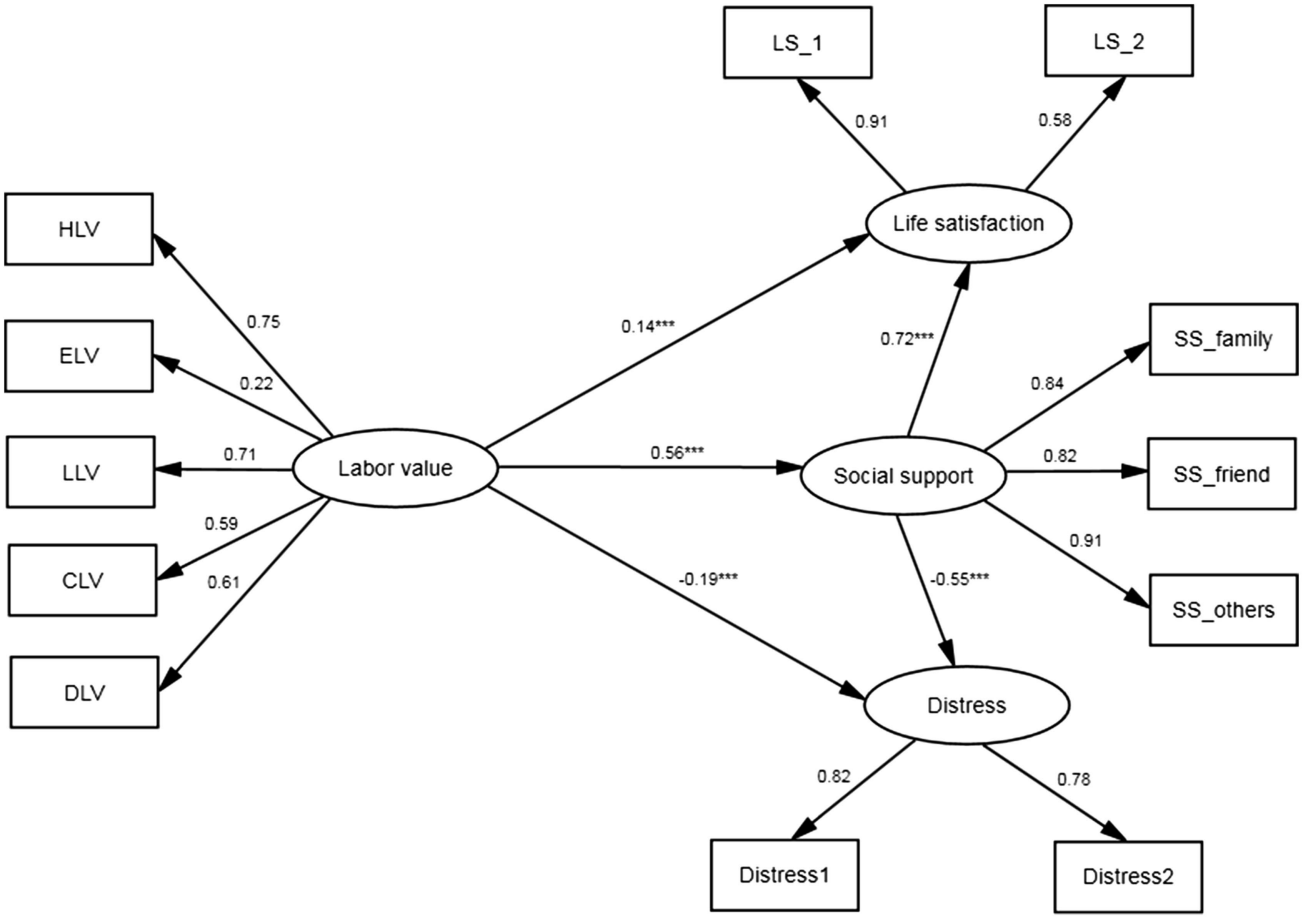
HLV, honest labor value; ELV, equal labor value; LLV, love labor value; CLV, cherishing labor value; DLV, distribution labor value; LS, life satisfaction.

Firstly, labor values can directly predict more life satisfaction significantly (*β*=0.61, *p*<0.001) while negatively directly predict less psychological distress significantly (*β*=−0.57, *p*<0.001). Then, we set up a structural model (model 1) with social support as a mediator, which has showed that the fitting degree of model 1 was good [*χ*^2^(49, 2691)=810.70, *p*<0.001; RMSEA=0.076; SRMR=0.045; CFI=0.9500]. Therefore, we used model 1 as the structural model of this study (see Figure 1).

In addition, the significance of the mediating effect in the model was assessed by using bootstrap estimation procedure, with 1000 bootstrap samples randomly extracted from the original data set (*N*=2691). As expected, the results showed that social support played significant and independent mediating roles between labor values and life satisfaction (95% CI=0.3736 to 0.4365), as well as between labor values and psychological distress (95% CI=−0.3411 to −0.2719).

### Gender Difference

We also examined whether there existed gender differences. Firstly, we tested whether there were gender differences in the four latent variables. The results indicated that there were gender differences in labor values (*t*=−3.135, *p*=0.002) and psychological distress (*t*=−5.666, *p*<0.001), and the boy group had significantly higher labor values and psychological distress than girl group. But there were no gender differences in social support (*t*=−0.589, *p*=0.556) and life satisfaction [*t*(2691)=0.214, *p*=0.830]. Because of the gender differences we have found, multi-group analysis was employed to test the stability of the model.

We constructed two models to compare with each other to check whether there are gender differences among their pathways. As suggested by Byrne (2001), two models that keeping basic parameters (i.e., factor loadings, error variances, and structure covariances) equal were established. The first model allowed free estimations of the path coefficients between females and males (unconstrained structural paths) and the second constrained all path coefficients to be equal (constrained structural paths). Chi square differences [△*χ*^2^(5, 2691)=15.49, *p*=0.008] between the two models were significant, suggesting the structural model was not stable across gender. What’s more, the critical difference ratio (CRD) was used to further determine whether there was a significant gender difference in each pathway. An absolute value of CRD greater than 1.96 indicated a significant difference between the two parameters. The results showed that three path coefficient was significantly different (CRD_LV→SS_=2.35, CRD_SS→PD_=−2.79, CR_SS→LS_=2.73). As to the pathway of LV→SS, the pathway parameter of boys (*β*=0.55) is less than that of girls (*β*=0.57), which means that compared with boys, girls with positive labor values can perceive a higher level of social support. Then for the pathway of SS→PD, the pathway parameter of boys (*β*=−0.49) is more than that of girls (*β*=−0.60), which means that girls with more social support can perceive a less psychological distress than that of boys. And for the pathway of SS→LS, the pathway parameter of boys (*β*=0.12) is smaller than that of girls (*β*=0.17), which indicated that the girls perceiving more social support would have higher life satisfaction.

The differences between boys and girls in labor values and psychological distress may be related to school education and family education. Although China has made remarkable achievements in advancing gender equality, people still have more expectations for men in terms of social responsibility. Therefore, in the family education and school education of minors, boys are educated to take responsibility and be brave. In the education of labor values, boys face more expectations. This may cause some psychological distress to boys.

## Discussion

This study explored the positive and negative effects of labor values on mental health from the aspects of life satisfaction and psychological distress, and further verified the mediating role of social support. The results showed that positive labor values can significantly promote individual life satisfaction and restrain psychological distress. Meanwhile, social support plays a significant mediating role in the relationship between labor values and mental health.

First of all, labor values are closely related to individual life satisfaction and psychological distress. On the one hand, the more positive the individual’s labor values are, the higher the individual’s life satisfaction is, which is consistent with the previous conclusions that positive values can promote individual life satisfaction (Lim et al., 2020; Ma and Ding, 2020). As an important aspect of individual value system, labor value can also positively predict individual life satisfaction. It is important for educators to provide students with values education in teaching positively to help students establish correct labor values, and then to improve students’ life satisfaction. On the other hand, labor values are negatively correlated with individual psychological distress. That is, labor values can significantly inhibit psychological distress. The more positive the individual’s labor values were, the less likely he was to feel psychological distress, which was basically consistent with previous research conclusions (Deci and Ryan, 2000; Yang et al., 2019; Chao and Wang, 2020). There are many factors that may affect individual mental health, and the labor value is an important factor to which educators need to pay attention, but it is often overlooked. Therefore, both parents and educators should pay special attention to cultivating children’s labor values when guiding them to maintain a positive and healthy psychological status. No matter in daily family education or school education, children should be guided to participate in the process of labor practice, which helps them establish positive labor values, then improves their life satisfaction, and protects them from negative effect of psychological distress (Liu et al., 2018).

Secondly, social support plays an important mediating role in the relationship between labor values and mental health in the aspect of psychological distress and life satisfaction. On the one hand, social support mediated the relationship between labor values and life satisfaction. That is, the more positive the individual’s labor values are, the higher the level of social support they feel from others around them, and the increased social support from others could further promote individual life satisfaction. Although there are few existing studies that directly discussed the effect of labor values on individual life satisfaction through social support, Feng et al. (2012) have found that individual internal traits could affect their life satisfaction through social support. Moreover, existing studies also supported the influence and positive predictive effect of values on social support (Constantine, 2006; Bubic and Erceg, 2018). As an important content of individual internal traits, labor values have a positive impact on individual life satisfaction through the mediating effect of social support. What should be noticed in this study is that there are gender differences when labor values influence individual life satisfaction through social support. It is showed that, compared with boys, the girls with higher perceived social support could be more satisfied with their life.

On the other hand, social support mediated the relationship between labor values and psychological distress. That is, the more positive the students’ labor values are, the higher social support they perceived from others, then the students with higher social support could feel less psychological distress. Previous studies on the influence of values on individual mental health through social support have figured out that positive values can make individuals perceive more social support from others around them. For example, Xiang et al. (2018) have found that gratitude can significantly predict more individual social support, that is, cultivation of individual gratitude can enable individuals to obtain more social support. Another study on secondary vocational school students also proved that positive internal qualities can significantly positively influence the mental health of students through perceived social support (Yuan et al., 2021). The results of this study are similar to the above research, that is, labor values can inhibit psychological distress through the mediation of social support. Our study further found that there were significant gender differences in the internal mechanism of labor values inhibiting psychological distress through the mediation of social support. Girls with positive labor values reported higher social support than boys, while boys with higher perceived social support can inhibit psychological distress more effectively than girls. This reminds educators and parents to pay special attention to the psychological pressure that boys may face when educating children on labor values. Labor values education and mental health assessment should be carried out at the same time, especially in school education. We need to maintain a balance between education on labor values and maintaining a healthy mental state. Therefore, an appropriate mental health protection plan for boys may be necessary while carrying out labor value education.

Based on the background of state policy which attaching more importance to the development of labor education, it is the first study to explore the impact of labor values on mental health and its internal mechanism from the perspective of social support theory, which has its significance in both reality and guiding practice. Specifically, it provides reference for primary and secondary school to practice labor education, helps the students to set up more positive labor values, and helps to promote their development of mental health. However, this study still has some limitations. For example, because the cross-sectional study design was adopted in this study, whether the conclusions can be extended to other age groups need to be further tested. So future studies can do research on different age groups and design longitudinal studies to explore the causal relationship of different variables. In addition, since the data used in this study are all self-reported, some errors may happen due to the interference of various situational and social factors. Therefore, future studies can carry out different ways of data collection to improve the objectivity of measurement.

Moreover, since our research is an explanation of the role of labor values in China’s special cultural background, labor values have special cultural significance. In Chinese institutional culture guided by Marxism, labor values have special meanings. In other countries, especially western countries, do labor values have other special connotations different from those in China? Is there a possible difference between the mechanism of labor values on mental health and the research based on Chinese samples? In the future, the above conclusions can be verified through cross-cultural comparative analysis.

## Data Availability Statement

The original contributions presented in the study are included in the article/supplementary material, further inquiries can be directed to the corresponding authors.

## Ethics Statement

The studies involving human participants were reviewed and approved by Ethics Committee of Hunan Normal University. Written informed consent to participate in this study was provided by the participants’ legal guardian/next of kin. Written informed consent was obtained from the individual(s), and minor(s)’ legal guardian/next of kin, for the publication of any potentially identifiable images or data included in this article.

## Author Contributions

All authors listed have made a substantial, direct, and intellectual contribution to the work, and approved it for publication.

## Conflict of Interest

The authors declare that the research was conducted in the absence of any commercial or financial relationships that could be construed as a potential conflict of interest.

## Publisher’s Note

All claims expressed in this article are solely those of the authors and do not necessarily represent those of their affiliated organizations, or those of the publisher, the editors and the reviewers. Any product that may be evaluated in this article, or claim that may be made by its manufacturer, is not guaranteed or endorsed by the publisher.
